# Supplementary material for: A cytokine panel and procalcitonin in COVID-19, a comparison between intensive care and non-intensive care patients
Source: PLoS One. 2022 May 2;17(5):e0266652. doi: 10.1371/journal.pone.0266652 (PMC9060342; doi:10.1371/journal.pone.0266652)
Supplement: Attachment — Submitted filename: Response to Reviewers .docx [file pone.0266652.s002.docx]

**Dear Editor**,

We would like to thank both reviewers for their very helpful comments and the Journal for offering us the opportunity to re-submit with revisions.

We have now addressed all the comments and our point by pint responses are as below. The changes made to the manuscript are indicated in red text.

Yours sincerely,

Tina

**Reviewer's Responses to Questions**

***Comments to the Author***

*1. Is the manuscript technically sound, and do the data support the conclusions?*

*The manuscript must describe a technically sound piece of scientific research with data that supports the conclusions. Experiments must have been conducted rigorously, with appropriate controls, replication, and sample sizes. The conclusions must be drawn appropriately based on the data presented.*

*Reviewer #1: Partly*

*Reviewer #2: Partly*

*2. Has the statistical analysis been performed appropriately and rigorously?*

*Reviewer #1: I Don't Know*

*Reviewer #2: I Don't Know*

*3. Have the authors made all data underlying the findings in their manuscript fully available?*

*The PLOS Data policy requires authors to make all data underlying the findings described in their manuscript fully available without restriction, with rare exception (please refer to the Data Availability Statement in the manuscript PDF file). The data should be provided as part of the manuscript or its supporting information, or deposited to a public repository. For example, in addition to summary statistics, the data points behind means, medians and variance measures should be available. If there are restrictions on publicly sharing data—e.g. participant privacy or use of data from a third party—those must be specified.*

*Reviewer #1: Yes*

*Reviewer #2: Yes*

*4. Is the manuscript presented in an intelligible fashion and written in standard English?*

PLOS ONE does not copyedit accepted manuscripts, so the language in submitted articles must be clear, correct, and unambiguous. Any typographical or grammatical errors should be corrected at revision, so please note any specific errors here.

*Reviewer #1: Yes*

*Reviewer #2: No*

*5. Review Comments to the Author*

*Please use the space provided to explain your answers to the questions above. You may also include additional comments for the author, including concerns about dual publication, research ethics, or publication ethics. (Please upload your review as an attachment if it exceeds 20,000 characters*

**Reviewer #1: Some suggestions:**

Abstract:

- in general, the "intensive care unit" is referred to the "ICU". Many health care system will also indicate whether the patient will be managed in the different specific ICUs, like medical ICU (MICU) or surgical ICU (SICU), but typically we refer to this level of care as an ICU. In the UK, where this study was done, ITU refers to the "intensive therapy unit" which from what I am reading is a direct correlate to the ICU as described in areas of the world like the United States. Since this publication is likely going to reach a global audience, I would suggest using ICU throughout the manuscript but if ITU is kept then the abbreviation should be placed in the title and "intensive therapy unit" should be used throughout.

**Response:** Thank you for the suggestion. We have revised the manuscript to the preferred terminology for intensive care, ICU throughout the article.

Methods:

- Line 122: please indicate how many patient beds are available at the hospital and the exact name of the Hospital where the study was conducted.

**Response:** We have now included the name and bed capacity of our hospital – lines 121 -122.

- In the methods section, you should include what were the general requirements for those admitted to the ICU at your hospital. For example, what level of oxygen supplementation was the minimum requirement to be admitted?

**Response:** The admission to ICU was considered when the oxygen requirement of the patient was 40% (10L) or more or those who show a rapid increase in oxygen requirement – lines 155-156.

Were these all patients placed on mechanical ventilation? I would include that in your methods and would even consider to include in the analysis.

**Response**: All ICU patients were receiving mechanical ventilation. Included in method section­­– lines 156-157.

- a small flow diagram of those included/excluded and reasons would be useful for the readers

**Response:** We have now included the flow diagram as suggested – figure 1.

- From the 63 age-matched non-ITU patients, how did you make that match? Did you match other chronic conditions? There could be a sampling bias at hand but this should be discussed.

**Response:** We only matched the groups for age. Chronic conditions were not matched between ICU and non-ICU patients. However, the common chronic conditions like Diabetes, Hypertension, lung disease, chronic kidney disease and cardiovascular disease between two groups were similar and have now added in results section Table 2, Page 11.

- Do we know who was on glucocorticoids and who was not?

**Response:** This has now been included in lines 166-167. A new Paragraph has now added in Results section because fewer patients in ICU were on glucocorticoids compared to non-ICU patients–lines 281-287.

- Do we know which SARS-CoV-2 variant was circulating in this regions during the study period? I would include that.

**Response:** This has now been included in lines 127-129.

Results:

- I think it is very important to discuss the clinical factors of those with and without COVID-19 as it relates to the cytokine and CRP testing; it is great to compare to healthy volunteers as a control but were they tested for COVID-19? Could they have been asymptomatically infected? Please explain the recruitment of the healthy volunteers.

**Response:** Details around the recruitment of the healthy volunteers has now been included – lines 149-156.

- Common comorbidities to consider in the analysis would be: Diabetes Mellitus, Hypertension, Obesity, Chronic Kidney Disease, Chronic Obstructive Pulmonary Disease, and others processes.

**Response:** This has now been included in results-line 247-251 and Table 2.

Discussion:

- I would add some discussion on IL-6 and CRP in the clinical context of COVID-19 and initiation of certain immune-modulating medications, like tocilizumab, or barcitinib, among others, who are found to have high initial CRP and IL-6. In the U.S., guidelines are now suggesting use in certain populations who have CRP greater than or equal to 75 mg/L and rapidly increasing oxygen requirements. I would also cite a large trial like the RECOVRY trial which was conducted in the UK. NHS also recommends IL-6 inhibitors in certain populations. A discussion on the results as it pertains to clinical use is suggested.

**Response:** We have now included the conclusions of the RECOVERY trial and NICE guidelines recommendations for Tocilizumab–lines 391-399.

Limitations needs attention. Do we know who was glucocorticoids (I would not use steroids).

**Response:** 75% of patients (ICU & non-ICU) were on glucocorticoids as part of the management of COVID-19 and associated complications. This has now been included in the manuscript –lines 166-167 and lines 281-287.

Small sample size is a big limitation and needs to be acknowledged.

**Response:** We have included the small sample size as a limitation of this study. The numbers however represent a high percentage of patients to have had cytokine results vs. other hospitals in the UK treating large number of COVID-19 patients. This is due to the limited availability of routine cytokine measurement in UK labs.

What does "some patients were requiring organ support" mean? This is why it is important to assess how patients were matched, what level of ICU care was needed and comorbidities of the cohort.

**Response:**

All ICU patients required mechanical ventilation, which is now included – lines 159-160.

Co-morbidities between ICU/non-ICU groups is now included in table 2 and lines 247-251.

**Reviewer #2:**

This article discussed relationships between cytokines, procalcitonin, and C-reactive protein among COVID-19 patients in London during certain months of the pandemic. The authors compared cytokines, PCT, and CRP between COVID-19 patients and healthy volunteers and also between ITU patients and non-ITU patients. The authors suggest previous research with cytokines to help determine any difference between viral infection and bacterial infection. The authors also admit that further research is needed to assess the relationship between findings from a cytokine panel and COVID-19.

Investigation of the inflammatory response among COVID-19 patients, such as those in an ITU setting, is important for clinical practice. While the intentions of this study are valid, it is difficult to follow the main aim and resulting comparisons included in the analysis. The Methods need to be clearer, especially with defining the study population and inclusion terms, and the overall organization of the paper needs improvement.

**Response:** We thank the reviewer for the above comments. We have now re-structured the manuscript to address this and hope it now provides better clarity.

**Major Comments:**

In the Introduction, include a description or paragraph about the “recently validated cytokine panel” as stated in lines 115-116. Give more background information on this panel, as this cytokine panel is one of main topics of this paper.

How was this particular panel validated?

How was this panel used previously? Briefly, how does this panel work?

**Response:** We have now expended on the cytokine panel to clarify when it was introduced and the process uses to validate its routine use – Lines 109-115. More details about validation mentioned in Method section under Cytokine Panel Performance Characteristics *-* Lines 182 -208.

The aim of the paper states that there were assessments of concurrent bacterial infections with COVID-19; however, there is a lack of descriptions about concurrent bacterial infections. In the Methods section, include how bacterial infections were assessed.

Which bacterial testing was used? Which bacteria could be detected on these tests? When were patients included in this study tested for concurrent bacterial infection?

**Response**: This is now included in method section (Lines 163-165) and in result section (lines 305-308).

In the Discussion, it is unclear how the findings are directly related to the main aim of assessing bacterial co-infection.

**Response**: we have now simplified the aim of this study (Lines 117-119) as our data is observational for bacterial co-infection. Described in the discussion–Lines 359-385.

The Methods and Results sections include comparisons with 24 healthy volunteers; however, it is unclear how the volunteers were chosen for this study.

How were these volunteers chosen?

Were these volunteers matched at all (the non-ITU patients were age-matched)?

When were these volunteers sampled? Were the volunteers sampled during the same timeframe as the other patients?

Describe the criteria needed to be considered “healthy.”

**Response:** The recruitment of the healthy volunteers has now been included – lines 149-156.

The volunteers were younger than the COVID-19 patients which is now included in result section (lines 221-222) and limitations (lines 414-415).

All volunteers sampled in April 2020 for the validation of the cytokine panel within our laboratory (line 151). A proportion of patients’ samples were collected during this period.

Also, the Introduction sets up the study to compare ITU and non-ITU patients, so some explanation about the COVID-19 patients versus “healthy” volunteers is needed earlier in the paper.

**Response:** We have now referred to the healthy volunteers in the abstract (line 47 and 51) and under Introduction (line 119).

Were comparisons made between ITU patients, non-ITU patients, and “healthy” volunteers? A three-way comparison with “healthy” volunteers as the referent may be more elucidating.

**Response:** We have now compared three groups as suggested (lines 229-232) and results are summarised in Table 1

**Minor Comments:**

Line 87: Make a new paragraph for CRP descriptions.

**Response**: CRP description is now in separated paragraph - Lines 84 – 88.

Lines 111-113: Clarify which study is referenced here and describe the control group used in that study. Introduce the study more clearly earlier in this paragraph.

**Response**: Reference given and control group is described - Lines 105-108.

Lines 117-118: Give some context about COVID-19 incidence in London during the study period and how the cytokine panel was used at this particular hospital. Give the months/year of the “peak” pandemic time.

**Response**: Included in the methods (Lines 125 -129), Introduction (line 109 to 115). ‘Peak’ pandemic time has been omitted from the Introduction as not relevant to our study data.

Line 122: State the specific months/year that included the patients in this study.

**Response**: March 2020 to January 2021, included in the methods (Lines 125-126)

Line 123: When were patients tested for SARS-CoV-2? Was there a specific timeframe from exposure that these patients were tested? Was the testing implemented for diagnostic purposes or as a general hospital screening test during that time period?

**Response**: The reason for admission of all patients in this study was suspected COVID-19 infection based on clinical/radiological evidence which was confirmed with positive (RT-PCR) SARS-CoV-2. Included in method section (Lines 137-139).

Line 127: Specify how many of the 75 excluded patients were ITU or non-ITU.

**Response**: 55 ICU and 20 non-ICU, now included in Figure 1.

Line 130: Explain why patients needed to have a same-day PCT result as the cytokine panel to be included in the study. Give the number of patients excluded based on not having same-day tests—was it 14 patients (make sure that the total numbers reflect the 76 included patients)?

**Response**: Included in line 143 in method and Fig 1.

Consider making a flow chart delineating the inclusion/exclusion factors for included patients.

**Response**: We have now included a figure to highlight this (fig 1, page 6).

Lines 142-144: Is this the test that was mentioned within the introduction?

**Response**: No in these references, they have used different methods for PCT.

Line 152: Spell out CV when it is first mentioned.

**Response**: Amended as suggested–Line 185.

Table 1: The row for PCT is missing. Add PCT results.

**Response**: PCT is not included in Table 1 as PCT was not measured in healthy volunteers. PCT is however included In Table 3.

Line 211: Indicate that the finding was statistically significant.

**Response**: we have made this change (Line 263).

Line 247: Interleukin is misspelled.

**Response**: This has now been corrected (Line 300).

Within the Discussion, explain why correlations were used for PCT and CRP among the ITU and non-ITU patients. Even though the correlations are statistically significant, most correlations seemed weak. How do these findings translate to clinical practice?

**Response**: We have suggested that correlation of cytokines with CRP and PCT may help to assist in identifying bacterial infections. Included under in discussion, lines 361 -363, 370-371, 375-376). We agree with the reviewer that the correlations are weak and larger studies are required to better assess this.

Line 269: How was “severity” defined? How can these higher cytokines be shown with certain clinical signs in patients?

**Response**: In general ICU patients with elevated PCT had higher TNFα and longer ICU length of stay. (Severity has been replaced by ICU length of stay- lines 330-333)

Line 289: Add an in-text citation.

**Response**: This has now been included–line 351 and 352.

Lines 291-292: How were concurrent bacterial infections confirmed in this reference study and also in the present study?

**Response**: In our study, concurrent bacterial infection has changed to concurrent and/or super added bacterial infection. Concurrent and/or super added bacterial infection were identified based on positive blood or respiratory cultures within 48 hours from cytokine panel request time. This is included under Methods - lines 163-175

In reference study, authors suggested significant increase in PCT could possibly reflect bacterial coinfection in those developing severe COVID-19 thus contributing to complicate its clinical picture, this is now included in lines 352-353)

Line 304: Add an in-text citation.

**Response**: Added-line 367.

Lines 311-314: Please re-phrase this sentence, as it is difficult to follow.

**Response**: This sentence has now been rephrased as requested–Lines 373-378.

Line 315: Are the “two groups” mentioned here the high and low PCT groups?

**Response**: Yes, now Corrected for clarity–Line 379.

Line 328: Please specify the “two values.”

**Response**: The two values were CRP and IL-6. We have now stated this as suggested–line 402.

Line 355: State the number of patients (46) instead of “good number.”

**Response**: This has now been corrected.

There are many typographical errors, such as missing hyphens and inconsistent hyphenation, missing commas, missed spacing between words, missing articles before nouns, and missing or incorrect punctuation.

**Response**: We have reviewed the entire manuscript and corrected as relevant.

6. PLOS authors have the option to publish the peer review history of their article (what does this mean?). If published, this will include your full peer review and any attached files.

If you choose “no”, your identity will remain anonymous but your review may still be made public.

Do you want your identity to be public for this peer review? For information about this choice, including consent withdrawal, please see our Privacy Policy.

Reviewer #1: No

Reviewer #2: No
