# Supplementary material for: A cytokine panel and procalcitonin in COVID-19, a comparison between intensive care and non-intensive care patients
Source: PLoS One. 2022 May 2;17(5):e0266652. doi: 10.1371/journal.pone.0266652 (PMC9060342; doi:10.1371/journal.pone.0266652)
Supplement: Attachment — Submitted filename: Response to reviewers .docx [file pone.0266652.s003.docx]

**Dear Editor,**

Thank you for offering us the opportunity to re-submit the manuscript with revisions.

We have now addressed all the comments and our point-by-point responses are as below. We hope this revised manuscript now meets the expected standards for publication.

All edits to the manuscript has been made using red text.

**Comment:** *Please cite the top-tier papers.

#1. Yang JM, Koh HY, Moon SY, Yoo IK, Ha EK, You S, Kim SY, Yon DK, Lee SW. Allergic disorders and susceptibility to and severity of COVID-19: A nationwide cohort study. J Allergy Clin Immunol. 2020 Oct;146(4):790-798. doi: 10.1016/j.jaci.2020.08.008. Epub 2020 Aug 15. PMID: 32810517; PMCID: PMC7428784.
#2. Shin YH, Shin JI, Moon SY, Jin HY, Kim SY, Yang JM, Cho SH, Kim S, Lee M, Park Y, Kim MS, Won HH, Hong SH, Kronbichler A, Koyanagi A, Jacob L, Smith L, Lee KH, Suh DI, Lee SW, Yon DK. Autoimmune inflammatory rheumatic diseases and COVID-19 outcomes in South Korea: a nationwide cohort study. Lancet Rheumatol. 2021 Oct;3(10):e698-e706. doi: 10.1016/S2665-9913(21)00151-X. Epub 2021 Jun 18. PMID: 34179832; PMCID: PMC8213376.

**Response:** The above two references have now been included under ‘Discussion’ – reference number 19 and 20.

**Comment:** There are numerous misspellings/typographical errors. Thorough proofreading of phrases is needed.

**Response:** Thank you for highlighting this. We have reviewed the manuscript and made relevant corrections throughout.

**Comment:** The terminology of co-infection, superadded infection, superinfection needs to be clearer. If the intent is for the same meaning across the manuscript, there needs to be consistency with the appropriate term. The nuances of these terms may need to be defined in the manuscript if the intent is for a molecular-based definition.

**Response:** Concurrent bacterial infection and co-infection is now replaced with

super-infection throughout because all confirmed bacterial infection in this cohort, occurred ≥48 hours after admission to hospital for COVID-19. (Lines 170-172)

**Comment:** Lines 111-112: At the very least, cite the published literature concerning the cytokine panel creation. It is unclear if this particular cytokine panel is location-specific or more widely used.

**Response:** Two additional references cited (Line 112). In addition the following sentence is now included in the manuscript - ‘While IL-6 is widely being used in the UK, this panel is unique to King’s College Hospital’. (Lines 111-116)

**Comment:**Line 149: Which co-morbidities were defined as absent from “healthy” volunteers?

**Response:** The sentence has now been re-structured for better clarity - Lines 153-155.

**Comment:**Methods: Which specific bacterial infections were included in the testing?

**Response:** We have now included the details of the culture results - Lines 310-317.

**Comment:** Methods: There are discrepancies between the number of patients in certain groups and the numbers listed in Figure 1. Please review and correct the patient numbers.

**Response:** The patient numbers have been corrected as relevant - Line 138.

**Comment:** Discussion: The discussion states that there are few limitations, but that is not accurate. As a retrospective study, there are several inherent limitations. Also, healthy volunteers were not tested for COVID-19 (as explained in the Methods), but this is still another limitation due to lack of confirmation.

**Response:** This has now been addressed – Lines 432-436.

Yours sincerely,

Tina Mazaheri
